# PICALM-MLLT10融合基因阳性伴多基因突变儿童T淋巴母细胞淋巴瘤/白血病1例

**DOI:** 10.3760/cma.j.121090-20231012-00195

**Published:** 2024-04

**Authors:** 妍 孙, 健 姜, 立荣 孙, 飞宇 闫, 玲珍 王

**Affiliations:** 青岛大学附属医院儿童血液肿瘤科，青岛 266000 Pediatric Hematology and Oncology Department, the Affiliated Hospital of Qingdao University, Qingdao 266000, China

患儿，男，12岁。因“发现皮肤出血点7 d，双下肢水肿3 d”于2023年5月5日入青岛大学附属医院。7 d前无明显诱因出现皮肤出血点，以腹部为主，伴全身乏力，无发热、牙龈出血、全身疼痛、视物模糊、全身水肿，未予特殊处理；3 d前出现双下肢水肿，伴全身多处皮肤瘀斑及阴囊肿胀，完善血常规：WBC 68.39×10^9^/L、ANC 9.64×10^9^/L、RBC 4.01×10^12^/L、HGB 126 g/L、PLT 117×10^9^/L。既往史、个人史、家族史无特殊。查体：身高138 cm，体重46 kg，体温36.5 °C，心率88次/min，呼吸24次/min，血压114/73 mmHg（1 mmHg＝0.133 kPa），神志清，精神欠佳，全身多发瘀斑，浅表淋巴结无肿大，心肺听诊未及异常，腹软，无压痛，肝脏肋缘下6 cm、剑突下7 cm，脾脏肋缘下5 cm，均质韧，无触痛，双下肢及足背部凹陷性水肿。

完善辅助检查：血常规：WBC 56.13×10^9^/L、ANC 6.30×10^9^/L、淋巴细胞计数46.95×10^9^/L、RBC 4.05×10^12^/L、HGB 123 g/L、PLT 110×10^9^/L、C反应蛋白1.28 mg/L。生化全套：LDH 425 U/L、ALT 56 U/L、AST 55 U/L、铁蛋白99.72 µg/ml、血沉8 mm/h。血凝常规：PT 14.3 s、APTT 39 s、TT 21.8 s、纤维蛋白原1.62 g/L、D-二聚体2 780.00 ng/ml。EBV-DNA及巨细胞病毒DNA阴性。消化系统超声：右肝最大斜径16.2 cm，左肝前后径10.0 cm，上下径11.4 cm，肝右前叶见2.2 cm×0.4 cm强回声；脾脏厚径6.1 cm，长径20.0 cm，内回声均匀，未见明显占位；脐周肠系膜根部见数枚肿大淋巴结，较大者1.3 cm×1.0 cm，髓质欠清；可见腹水。胸部CT：肺内未见明显实质性病灶，双侧腋窝多发肿大淋巴结。腹部、盆腔CT：肝脾增大，肝内钙化灶，胆囊萎缩、壁增厚，腹腔及腹膜后多发肿大淋巴结，腹腔少量积液；双侧髂血管走行区及腹股沟多发肿大淋巴结。颅脑MR：颅骨板障DWI高信号。

初诊骨髓形态学：淋巴细胞比例约占51.0％（大部分淋巴细胞胞体较小），可见约6.0％的幼稚淋巴细胞。诊断：慢性淋巴细胞白血病？骨髓免疫分型：70.55％的异常T淋巴细胞，表达CD45（+）、CD7（+）、CD34（−）、CD38（dim）、CD99（+）、cyCD3（+）、sCD3（dim）、CD5（dim）、CD2（+）、CD4（−）、CD8（−）、cyTdT（−）、CD1a（−）、CD56（−）、CD117（−）、CD33（−）、CD13（−）、CD19（−）、CD79a（−）、cyMPO（−）、TCRγ/δ（+），其中20.56％的异常T淋巴细表达CD99（++），免疫表型倾向幼稚欠成熟阶段。RNAseq结果示PICALM-MLLT10融合基因阳性，SMC3基因上检测到移码突变：c.2250_2251del(p.Lys751AsnfsTer5)；JAK1基因上检测到错义突变：c.2108G>T(p.Ser703Ile)。骨髓TCR基因重排：检测到TCRβ、TCRγ和TCRδ基因发生重排。骨髓染色体核型：47,XY,+X[2]/46,XY[28]。骨髓活检：苏木素-伊红（HE）染色及过碘酸-雪夫染色（PAS）示骨髓增生极度活跃（细胞容积80％～90％），可见一类细胞弥漫性增生，细胞中等大小，细胞核不规则，结合免疫组化结果，以T淋巴细胞成分为主，比例约为70％，诊断意见为T淋巴细胞增生性病变，倾向T淋巴母细胞淋巴瘤/白血病（T-LBL/ALL）；免疫组化：CD3（+）、CD7（+）、CD20（个别+）、PAX5（个别+）、CD10（−）、TDT（少量弱+）、MPO（粒系+）、CD5（−）。特殊染色结果：普鲁士蓝（−），网状纤维（骨髓纤维化：2级），Masson（胶原化分级：0级）。

颈部右侧淋巴结穿刺活检示淋巴组织增生性病变，免疫组化：TdT（+）、CD99（+）、CD38（−）、CD4（−）、CD8（−）、CD2（+）、CD5（90％+）、CDla（−）、MPO（−）、CD117（−）、CD33（−）、Lys（−）、Ki-67（80％+）、EBER（原位杂交，−）。肝组织穿刺活检病理示部分肝组织，肝细胞水肿、淤胆，可见点灶状坏死，组织内见较多淋巴样细胞浸润；免疫组化：TdT（散在+）、CD4（−）、CD8（−）、CD99（+）、CD2（+）、CD5（+）、MPO（−）、CD117（−）、CD33（−）、Lys（−）。

综合诊断为“T-LBL/ALL（伴TCRγ/δ重排）”。按照T-LBL/ALL给予VDLD（长春新碱+柔红霉素+培门冬酰胺酶+地塞米松）方案治疗。化疗第15天，肝脾未回缩，复查骨髓形态学：淋巴系增生明显，成熟淋巴细胞占57％，以异常淋巴细胞为主，其胞体略大、类圆形、核质比增大、偶见核畸形变、胞质量极少，天蓝色；幼淋可见，占6％。骨髓微小残留病（MRD）：异常T淋巴细胞57.05％。化疗第17天加用环磷酰胺+硼替佐米化疗，化疗第25天骨髓穿刺评估，骨髓MRD示异常T淋巴细胞43.50％。化疗第33天行骨穿，骨髓MRD可见24.73％异常T淋巴细胞，查体肝脾仍肿大，复查PET-CT示颌下双侧、颈部双侧Ⅱ～Ⅴ区、双侧锁骨上窝（大者约8.0 mm×7.0 mm）、双侧腋窝（大者约7.5 mm×5.5 mm）、肝胃间隙、肠系膜区、腹膜后腹主动脉及下腔静脉周围（大者约11.0 mm×7.0 mm）、双侧髂血管走行区、双侧腹股沟（大者约8.0 mm×5.0 mm）多发大小不等淋巴结，较疾病初期检查缩小，颈部淋巴结部分代谢增高，最大标准摄取值（SUVmax）约3.8；以上考虑淋巴瘤治疗有效；肝大、脾大，未见异常代谢增高。综合评估结果提示患儿处于疾病部分缓解状态。化疗第42天，予维奈克拉+芦可替尼口服1周，化疗第49天骨髓MRD可见4.84％异常T淋巴细胞，PICALM-MLLT10基因转阴。化疗第50天继续维奈克拉+芦可替尼口服1周联合CAT（环磷酰胺+阿糖胞苷+巯嘌呤）方案化疗，肝脾回缩；复查骨髓MRD可见0.75％异常T淋巴细胞。其后再次予CAT方案化疗后，复查骨髓MRD可见0.10％异常T淋巴细胞。目前患者仍在进一步化疗中。

讨论：T-LBL/ALL占儿童ALL的10％～15％，虽然近年治疗方案的优化明显提高了T-LBL/ALL的预后，但难治及复发仍是难以解决的问题。淋巴瘤的异质性是导致诊断困难、治疗失败的原因之一，所以需要综合临床表现、免疫分型、病理及基因检测来明确诊断。本例患儿骨髓形态学考虑白血病，但具体类型难以确定。为进一步明确诊断，进行了免疫分型检测，骨髓免疫分型结果显示TDT阴性，TCRγ/δ阳性，并非为典型的T-LBL免疫分型；此外，患儿肝脾肿大明显，疾病初期考虑肝脾γ/δ淋巴瘤。该患儿淋巴结穿刺病理结果提示T淋巴细胞弥漫增生，表达TDT、CD99，支持为幼稚细胞来源，未见髓系表达，免疫表型特点不符合早期T前体细胞淋巴细胞白血病。肝脏穿刺组织中CD4和CD8均阴性，CD99阳性，部分细胞表达TDT，免疫表型与淋巴结中相似，免疫分型不支持肝脾T细胞淋巴瘤，支持为T-LBL，因此，综合各方面检查结果，最终诊断为T-LBL/ALL。

